# Health Communication Cards as a Tool for Behaviour Change

**DOI:** 10.1155/2014/579083

**Published:** 2014-02-06

**Authors:** Carrie L. Matteson, Thomas D. N. Merth, Diane T. Finegood

**Affiliations:** ^1^Department of Biomedical Physiology and Kinesiology, Simon Fraser University, WMC 2805, 8888 University Drive, Burnaby, BC, Canada V5A 1S6; ^2^Faculty of Medicine, University of British Columbia, The Gordon and Leslie Diamond Health Care Centre, 2775 Laurel Street, Vancouver, BC, Canada V5Z 1M9

## Abstract

Individuals seeking healthcare treatment in the context of obesity often experience difficulty engaging in discussions around their health and face challenges finding consensus with practitioners on care plans that best suit their lives. The complex set of biological, social, and environmental variables that have contributed to the higher prevalence of obesity are well illustrated in the foresight obesity system map. Effectively understanding and addressing key variables for each individual has proven to be difficult, with clinicians facing barriers and limited resources to help address patients' unique needs. However, productive discussions inspired by patient centered care may be particularly effective in promoting behaviour change. Tools based on systems science that facilitate patient centered care and help identify behaviour change priorities have not been developed to help treat adult obesity. This project created and pilot tested a card based clinical communication tool designed to help facilitate conversations with individuals engaged in health behaviour change. The health communication cards were designed to help direct conversation between patients and healthcare providers toward issues relevant to the individual. Use of the cards to facilitate patient driven conversations in clinical care may help to streamline conversations, set realistic care plan goals, and improve long term rates of compliance.

## 1. Introduction

Obesity is widely recognized as a complex and serious health problem, yet overweight individuals struggle with losing weight and maintaining weight loss. Health professionals often feel ill equipped to offer support that may lead to success for their clients, and attempts to engage patients in conversations about weight reduction have decreased even in patient populations with the most need [1]. Obesity rates are forecasted to increase as much as 33% in the next 20 years [2], potentially adding to an already high annual estimated health care cost of over $140 billion [3]. The current high rates of obesity have emerged from a complex set of biological, social, and environmental variables. The foresight obesity system map illustrates this complexity, demonstrating 109 variables and 304 connections between them [4]. These variables represent the multiple levels of factors associated with obesity, from population to individual level variables. To successfully combat obesity as a society, solutions will likely need to target all of these levels. However, clinical treatment strategies often focus on individual behaviour change, encouraging increased rates of physical activity and decreased caloric intake. Public health initiatives focus on altering environmental stimuli to reach the same end, but barriers such as policy resistance produce lacklustre results in shifting obesity trends.

Individuals commonly seek help for reducing their weight at commercial facilities such as weight loss and physical activity centers, in addition to physicians' offices. For any person seeking help with behaviour change, only a subset of the many individual level variables in the foresight map may be relevant. Effectively understanding and addressing this subset has proven to be difficult, with clinicians often reporting obesity treatment as being “doomed to failure” [5], frustrating, and ineffective [6]. This stance is problematic, as clinicians are able to positively influence patients' health related behaviour by providing patients with some form of behavioural counselling, especially when patients actively participate [7, 8]. Patient centered care may decrease health care costs, as one study found that participants who received patient centered care sought less diagnostic tests and requested less referrals and follow-up medical visits [9].

The Canadian Clinical Practice Guidelines on the Management and Prevention of Obesity provide some guidance to clinicians by addressing pharmaceutical, surgical, cognitive, and behavioural treatment or prevention strategies [10]. The behavioural and cognitive treatment of obesity is important as it is the least invasive, and it may be more successful than pharmacotherapy, especially in maintaining long term weight loss after the treatment has stopped [11]. Behavioural treatment can exist on its own, or as an important component of these other approaches [12]. Discussions inspired by patient centered care may be particularly effective in behaviour change [13]. However, a review of chronic disease care practices in 13 different countries revealed a lack of self-management support, especially in the realm of emotionally based care challenges [14].

Strategies for effective communication between patients and healthcare practitioners are a key element of behavioural treatment. Decision and communication support aids have been utilized in the treatment of other diseases such as cancer and diabetes to help patients understand treatment options and provide personalized care [15–17]. In the United Kingdom, diabetes cards were developed as part of a project to address the difficulty some patients had in articulating specific challenges in managing their diabetes. Cards consisted of statements constructed from written descriptions of the problems and healthcare needs of diabetes patients. In this way, patients were able to develop their own “diabetes care agenda” [16, 18]. No such systems science based tools that facilitate patient centered care and help identify behaviour change priorities have been developed to help treat adult obesity. Based upon the success of previous support aids and patient communication tools, we aim to generate a tool to guide individuals with health behaviour change.

This project created and pilot tested a card based clinical communication tool designed to help facilitate conversations with individuals engaged in health behaviour change. The health communication cards were designed to help individuals understand the broad range of variables implicated in obesity and to provide a simple tool for generating patient focused goals. The tool is designed to help direct the conversation between patients and healthcare providers to issues relevant to the individual, as opposed to a more generic and simplistic “one size fits all” discussion of the need for more exercise and less food intake. Use of the cards to facilitate patient driven conversations in clinical care may help to develop autonomy and self-motivation in behaviour change, which are shown to improve long term rates of compliance [19].

## 2. Methods

### 2.1. Statement Development for Health Communication Cards

Card statements were chosen to represent the broad range of factors implicated in obesity at the level of the individual while including cognitive and behavioural variables found to be important in obesity treatment. These issues were drawn from a system map illustrating the complex influences on obesity in addition to the clinically relevant cognitive and behavioural concepts identified in the treatment literature. The foresight obesity system map was used to construct cards describing individual level variables because it serves as a comprehensive overview of many different areas implicated in obesity with a complex systems lens [4]. All seven clusters on the map (food production, food consumption, individual physical activity, individual psychology, social psychology, physical activity environment, and physiology) were utilized to generate statements worded to describe a problem that an individual might face based on the variables illustrated in the map. For example, the variable “food variety” was converted to the statement “I do not try new foods often,” while the variable “cost of physical exercise” was converted to “Physical activity is too expensive.”

Specific cognitive and behavioural variables implicated in obesity and shown to be effective treatment targets were further explored and used to inform the development of the cards. These variables include eating self-efficacy [20, 21], exercise self-efficacy [22, 23], binge eating behaviour [23, 24], flexible cognitive restraint, disinhibition, and hunger [25–28]. Validated and widely used tools to assess these variables provided background for generating statements in these areas. For example, the weight efficacy lifestyle questionnaire assesses eating self-efficacy and has a series of statements that assess self-efficacy in situations where food is readily available. The card “I do not feel confident in my ability to resist overeating when many kinds of food are available (such as at a buffet)” was created to reflect this. The intention was to capture a full range of issues that may be relevant to individuals as they try to communicate the details surrounding their individual circumstances regarding health behaviour change. A total of 64 card statements were generated for the health communication cards.

### 2.2. Semistructured Interviews and Focus Group

**Figure 1:**
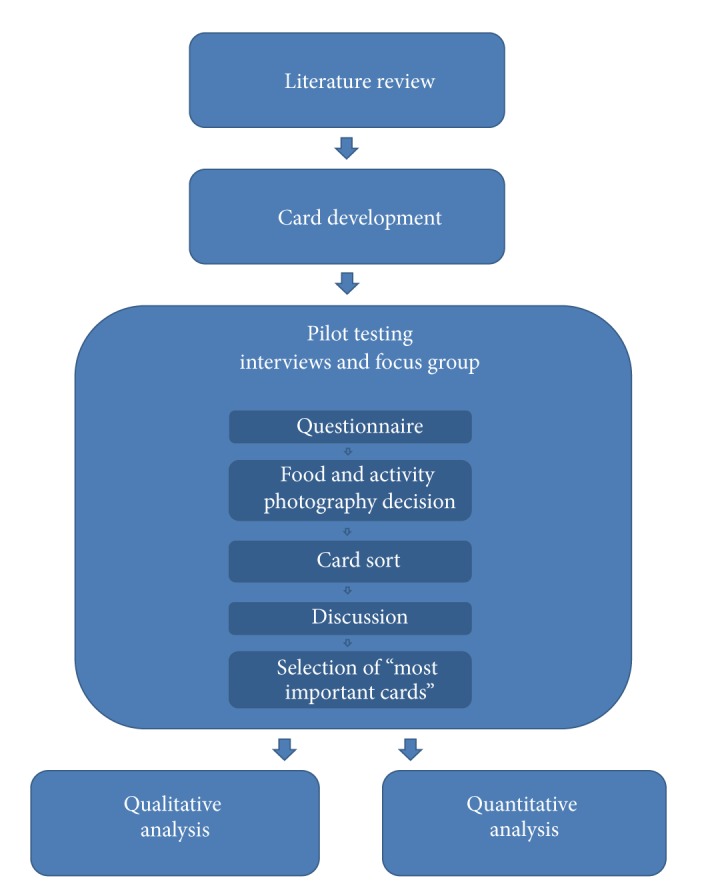


All subjects were actively trying to make changes in eating and exercise behaviours and were purposively recruited to participate in either semistructured interviews (*n* = 10) or a focus group (*n* = 8). A research design flow chart is shown in Figure 1. Ethical approval was obtained by the Simon Fraser University Office of Research Ethics. For interviews, two trained researchers worked with patients at an internal medicine specialist's office. Adult patients who were overweight or obese, were seeking treatment for a weight related condition, could speak and read English, and had an appointment during that time were eligible. The recruiting physician spoke to all patients seeking treatment for a weight related condition about the project, and interested individuals were directed to the on-site researchers. After obtaining signed, informed consent, participants contributed to individually recorded conversations regarding their challenges and successes with lifestyle change while sorting through the health communication card deck.

Insights gained through interview data analysis were used to modify the interview script in order to further explore targeted lines of inquiry during a focus group located in a different clinical context and geographic location. Participants were individuals participating in a weight loss surgery support group. The group was self-organized by patients who had undergone bariatric surgery some time in the past and included members who were waiting for surgery. The intent of the group is to provide treatment information, postoperative support, and guidance with health behaviour change to group members at any stage in the surgical continuum (from presurgical treatment to many years post-op). Support group members were comprised of adults who spoke English, were self-seeking support with regard to lifestyle change, and had access to bariatric surgery. The health communication cards project was introduced by the group founder and core facilitator at a meeting in advance of the focus group date. All individuals interested in participating were asked to attend the next regular meeting. After obtaining signed, informed consent, participants engaged in a focus group discussion about behaviour change and provided feedback regarding their experiences with the health communication cards. The discussion was facilitated by the same two researchers who previously conducted the individual interviews.

The protocols for interviews and the focus group were similar. Participants first completed a questionnaire to assess basic demographic and health information. As an opening exercise, participants were shown two sets of contrasting photographs. The first set of photographs displayed similar foods representing a weekly grocery shop, but one image contained more packaged foods while the other had more fresh and organic foods. The next set of images depicted leisure time activities (such as an exercise ball and active games) in contrast to outdoor sports equipment (such as skates and bikes). Participants were asked to choose which photos reflected healthy living and describe the types of families who might be represented by the images.

All participants sorted through their own deck of health communication cards and were instructed to categorize the cards into two piles: “cards that describe me” and “cards that do not describe me.” Conversation about the card sort activity followed, which included discussion around the best uses of the cards and exploration of which types of health workers or social supports participants felt would be most likely to engage in such an activity. Participants were prompted to select their three to five most important cards from the “cards that describe me” pile.

### 2.3. Quantitative Data Analysis

Demographics information in addition to card selection data were entered into Microsoft Excel 2010 and transferred to SPSS (PASW Statistics 18) for statistical analysis. Graphs and figures summarizing results were produced in both Excel and SPSS. Spearman's correlation coefficients were calculated for age and body mass index (BMI) as they relate to the number of cards selected in the first card sort.

A total of 18 participants completed questionnaires and the card sort activity. Both the interview and focus groups were similar in participant demographic profiles (Table 1). A greater portion of the interview sample participants were single (40%) and had at least one chronic disease (90%) compared to the focus group where no participants were identified as being single and fewer identified as having a chronic disease (37.5%).

### 2.4. Qualitative Data Analysis

Interpretive description is a qualitative methodology developed to investigate clinical phenomena. This methodology guided the research design, data collection, and analysis [29, 30]. Interview and focus group audio files were transcribed verbatim using Transcribe! transcription software (Seventh String software, Version 8.10, 2010). NVivo qualitative data analysis software (QSR International Pty Ltd. Version 8) was used to track coding themes. All data received multiple passes by two researchers for coding based upon themes generated inductively from the data. Interpretive description [31] served as the conceptual framework for analysis, allowing for a rich understanding of how and why participants may have felt and behaved as they revealed during discussions. Grounding for data display and knowledge development was based on a complex systems approach to obesity [32].

## 3. Results

### 3.1. Card Selection Trends

The mean number of cards selected as “applies to me” by any one participant during the card sort was 25.4 (the lowest 5, the highest 41) out of the total 64 health communication cards in the deck (Table 2). There was a significant (*P* = 0.01), negative correlation between age and number of cards selected with Spearman's correlation coefficient −0.58, explaining 34% of the variance in the relationship.

**Figure 2:**
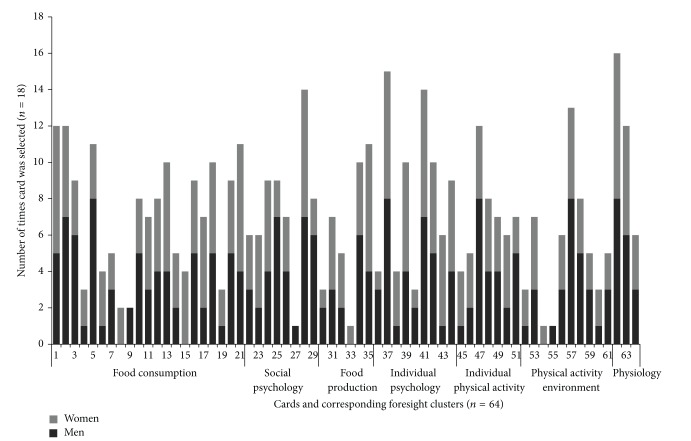


Most cards selected were not different between men and women, although there were several exceptions (Figure 2) in these participant groups. The most frequently chosen cards were not necessarily the ones that participants thought were most important to them. For instance, the two most popular cards in the general card sort were only selected by one participant when asked to narrow their general “applies to me” stack down to the most important cards (Table 3).

### 3.2. Eating Disorders and Body Dissatisfaction

No men and 44% of women selected the card “I do not pay much attention to changes in my figure,” while 56% of women and 11% of men selected the card “I regularly feel disgust, shame, or self-hate from overeating.” These selections may highlight some gender sensitivities regarding social norms around food and body image. It is striking that only women reported they did not pay attention to changes in their figure. However, two of the women who made that selection also chose “my body size and shape influence how I value myself,” reflecting the conflict and confusion experienced in our society while dealing with weight issues. This pattern of card selection may also be an attempt to clarify that, while they are affected by being overweight, they try not to obsess about their size while engaging in behaviour change. The paradoxical card selections claiming no concern about changes in physical shape while simultaneously reporting that body size influences self-value opens a door for productive conversations around body image sensitivities that may be difficult to broach or may not have come to light without use of the cards.

The cards “I eat way too quickly” and “I often eat too much food and feel uncomfortable” represented items from the binge eating scale [24] and were some of the most popular choices when participants selected cards that described them. Each of these statements were selected by 12 participants. Of the cards chosen most for “cards most important to describe me,” four listed feelings or behaviours associated with binge eating. All of these cards were chosen four or more times and represented themes involving weight and shape overvaluation, secrecy when eating, and perceived loss of control (Table 3).

The four most commonly selected cards included statements regarding body image and dieting behaviours, such as “I have tried dieting and/or weight loss medication” (selected by 16 participants), “my body size and shape influence how I value myself” (14 selections), and “I battle between trying to eat healthy and eating foods I like” (14 selections). These statements were selected equally between men and women.

### 3.3. Individual and Environmental Variables

Some of the card statements described a variable focused on the individual, while some described more environmental or ecological elements (Figure 2). Cards that described individually focused variables were more numerous and were selected more frequently within the psychology based set of statements. Many of the psychological variables were emotional in nature, such as “I do not feel confident in my ability to resist overeating when I am nervous, depressed, or angry.”

Individuals recognized both the societal and individual influences on obesity, as overall participants selected equally from both. Nearly all participants chose at least one card from all seven of the clusters represented in the foresight obesity map (Figure 2). However, when participants selected their most important cards, they focused on more proximal issues involving personal behaviours.

### 3.4. Desire to Connect, Acknowledge Success, and Make Meaning with Health Workers

Although not asked to do so as part of the card sort activity, many participants provided a personal narrative of the reasons behind their overweight or obesity. These reasons included discussions of work, the physical and social environment, childhood, family, and many factors outside their direct control. These stories may have been an attempt to resolve the internal conflicts individuals seemed to have regarding their struggle with weight, validate their experiences and connect with care providers.

One theme that was never specifically asked about but was mentioned by nearly half of the participants was a comparison of cards they would have picked in the past to the cards they currently picked as “applies to me.” Participants noted the following:

*It made me feel good…it made me realize how much I've changed and how much I've realized that there's some of those things that I do not have to worry about.*

*It made me realize how far I've come.*

Participants spontaneously created a third pile of cards which they said used to describe them but no longer did. There was a sense of pride with this group of individuals who seemed to have turned the corner on their battle with health behaviour change in some way, and the card sorting process was very validating for them. This finding suggests that reflecting on accomplishments around behaviour change when progress has been made can be a positive and motivating experience that may be beneficial for some individuals.

Sorting the cards with or prior to seeing a healthcare worker was generally viewed as something that would make for a better clinical visit. Participants expressed that the cards helped identify concerns and stated that this would be useful in helping stimulate thinking about what they wanted to talk about prior to meeting with the practitioner. Additionally, participants felt that use of the cards may help practitioners provide more thoughtful care instead of listing simplistic diet and exercise advice:

**…*it would be really great having this before, like you know, when I see [my doctor] because I'd know what to tell her, but it just [reminds] me what I really want to talk about.*

*I think it would be better because I would know specifically what to say…what really affects me…we could just focus on the main thing how I feel.*

*…it's gotta be better than going in blind and dealing already with those kinds of misconceptions, you know? Like the “oh, you should just eat less and exercise more”.*

### 3.5. Bias in Establishing Appropriate Behaviour Change Targets

A minority of participants initially did not believe that the cards would be helpful in communicating with a healthcare worker. However, once they went through the whole exercise and were asked to choose cards most important to them, they generally found the cards helpful in assisting them to prioritize their efforts in behaviour change:

* Avoiding generalities that are going to offend us more than help us. You told me I have to lose weight, yeah I did not know it, come on.*

There was total agreement with all participants that physicians were unlikely to spend the time to go through the cards and talk with patients at medical appointments, and specialists or other healthcare workers with the time and experience to engage in meaningful counselling with patients were perceived to be most likely candidates for use of the cards.

Though participants stated that the cards did not introduce any novel variables they had not previously considered, they did report that the cards were helpful in establishing goals and bringing issues top of mind. Both the interview and focus group participants had generally been struggling with obesity for quite some time. Many participants who had completed interviews had previously met with the internal medicine specialist to discuss obesity and related comorbidities, meaning they were integrated into the medical paradigm and likely had multiple interactions and discussions about weight issues. All but one participant in the focus group had undergone bariatric surgery some time in the past 10 years. Therefore, all participants were engaged in the medical model of obesity treatment and could envision how using the cards may impact their experiences with care providers.

Similarly, participants reported that the photograph activity did not provide any opportunity for novel health education. When asked to identify the more healthy photographs of food items and physical activity options, all participants immediately identified the photographs featuring fresh foods and active sports equipment as compared to more packaged foods and sedentary activities. There was not a moment of hesitation or confusion from any participant about the healthy choices, yet some participants reflected personal guilt with “should” statements or remarked about societal beliefs that obese individuals would be more likely to select the less healthy images depicted.

### 3.6. Using the Cards with Family or Friends

Participants had mixed feelings about using the cards with family and friends with a relatively even split between finding the cards useful in that context or not:

* I would be able to use it with my partner and I would be able to use it personally, probably we would do it together.*

*…it's hard, just sometimes they do not know like how I feel. And so maybe like showing them these cards would…show them “ok then, she is struggling.”*

* Well my family, no. Because my husband, 148 lbs and he's been that since grade 12. My daughter wears size 2 and 4 clothes. My son is normal but [lives far away]. For me, no, I do not bring myself out to my friends. I do not talk about that kind of thing with my friends really.*

Several participants became quite emotional at this stage of the discussion. For example, one participant reported a desire to express more confidence about addressing the issue listed on the card in an effort to appear more accomplished. This finding suggests that the cards helped bring the discussion towards very personal and sensitive issues and is not amenable to online use or some digital applications.

## 4. Discussion

### 4.1. Age, Frequency, and Prioritization

There was a negative correlation between age and number of cards selected, which may have been due to the fact that older participants had spent more years engaged in health behaviour change and had already dealt with more of the issues stated on the cards. The fact that younger participants identified with a higher number of card statements may reflect that they experienced, or more fully recognized, increased complexity in their situations. Other studies examining health behaviour change trends have also identified differences related to age, where younger participants were more likely to participate in a treatment program and recognize barriers to change [33, 34]. The most popular card choices were not necessarily the most important issues for everyone (Table 3). This indicates that although some card statements may apply to most patients seeking help with lifestyle change, they are not necessarily the most important when individuals critically consider what they want to change.

### 4.2. Body Image and Binge Eating Disorder

Research has shown that, in general, women are less satisfied with their bodies compared to men [35] and, unlike men, this lack of satisfaction may not be related to BMI [36]. Our finding that men and women demonstrated variation in card selection related to body image may be reflective of this, or of gender related levels of comfort with discussing body image issues.

Aspects of binge eating behaviour may frequently occur in individuals seeking treatment for obesity. Methods that assess level of binge eating frequently include behavioural, physical, and psychological variables [24, 37]. The two most selected binge eating cards may help identify participants who could benefit from support aimed at the behavioural elements of binge eating (e.g., rate of eating and physical discomfort following overeating). Furthermore, as psychotherapy for treatment of binge eating disorder is costly [38], addressing the behavioural components which may be more prevalent may be a cost-effective way that health practitioners can begin to address some binge eating characteristics in a productive manner.

While card selection patterns featuring body image valuation in parallel with not paying much attention to weight may seem paradoxical, ambivalence is a normal part of any type of attempted behaviour change. Card selection regarding body image may also be related to the well-documented social stigma that overweight and obese individuals experience in their day-to-day lives. Additionally, there is evidence that body weight and shape concerns decrease following bariatric surgery [39]. The women that selected both cards may have lost weight and were satisfied with their body weight and shape and no longer felt the need to monitor these variables as closely. Future use of the cards should explore user rationale for card selection, as this may provide important insight into the interaction between what determines individual differences in self-value and how those variables are dealt with or monitored by each participant.

Discussions around weight can be challenging and subsequently result in patients feeling shame, fear, and ambivalence. These feelings may contribute to apprehension in reporting binge eating behaviours, even when directly asked. Using the cards may not only help with screening for eating disorders, but the process may set the tone for a more gentle and supportive conversation about how patients experience their relationship with food and their own body image concerns. As opposed to a diagnostic tool that definitively categorizes patients, the cards are meant to open conversations about topics that warrant further exploration or follow-up.

### 4.3. Changing Paradigms and Building Trust in Patient Care

Although no longitudinal data were obtained in this study, many participants noted that they had lost a significant amount of weight and had made important changes to their behaviour. This was also evident from the comparison many participants made about their current card selections and how many more they would have selected in the past. There is evidence that patients who feel more educated on their disease and treatment have better outcomes [40]. In this case, many of the participants may have reached saturation such that any additional information about variables and factors implicated in their obesity was not perceived to be useful. Further research is required to determine whether patients who are just starting to become more educated on their obesity and health related behaviours would benefit from this type of education. Participants brought up the issue of having to “change everything,” noting that it is impossible to simply change one thing and that a total “change of attitude has to happen” to attain significant alterations in outcome. Changing one's paradigm may be the most effective but most difficult way to alter one's health related behaviour [41]. Behavioural interventions should focus on making sure patients understand that, although weight changes may be part of the end goal, their target weight may or may not be reached through their process of cumulative attempts to change individual behaviours. Most importantly, they need to understand that, even if weight goals are not attained, the changes made through engaging in more healthy behaviours will positively impact quality of life, health risks, and metabolic variables.

Participants expressed vulnerability when talking about several issues identified on the cards, which revealed various areas of sensitivity that may not necessarily come up during regular clinical visits. Participants seemed to adopt a coping mechanism of focusing on the seemingly more optimistic view of where they wanted to be in terms of their health behaviour change journey, as opposed to where they actually felt they were. Helping individuals self-reflect at this level can be a difficult, emotional, and uncomfortable experience. This process warrants that a high level of trust and comfort with the healthcare provider must exist before patients will open up and discuss the reality of their situation. This is a critical step for developing a relevant and realistic care plan that is truly patient centred. Patients report that increased physician caring, communication, and efforts to partner with them in generating care plans are important elements in building trust [42]. The card sorting process may help to surface latent concerns while building trust into the patient/provider relationship such that more challenging or ambiguous issues may be addressed respectfully over time.

Physicians may also have difficulty sorting through the complexity of the problem. Some participants noted that physicians gave them overly simplistic advice and avoided going into the details of their lifestyle challenges with them. There was frequent frustration that conversations with health providers were didactic and simplistic, suggesting that a conflict exists between overweight patients and the advice they receive from health practitioners. The overly simplistic advice participants often receive leads them to disregard clinical advice and to believe that the health practitioner does not understand their condition. If patients could present their most concerning health issues using cards as a vehicle to direct conversations, this may provide practitioners with a means of addressing obesity that is directly relevant to their patients without ignoring the complex nature of the problem. While no participant in this study hesitated to identify the photographs of the healthier food items and active supports as compared to the less healthy options (suggesting a clear understanding of healthy lifestyle choices), clinical advice often continues to instruct patients what to do (eat less, move more) rather than help them understand how to improve behaviours [43]. The cards help care providers avoid making the error of delivering simplistic information about what healthy behaviours are, and instead direct productive conversations that help patients decide how to best achieve their goals in the context of their lives.

### 4.4. Establishing Relationships and Goals

Without being explicitly asked, many participants gave detailed accounts of their perceived reasons for being overweight or obese, often in the form of a story. These may have been an attempt to resolve the internal conflict many individuals seemed to struggle with rationalizing between the individual and environmental causes of their health related behaviours. Many of the individually focused cards might have been perceived as putting themselves at fault, while the environmental variables may have alleviated guilt by surfacing contributing factors outside of personal control. This is consistent with other research which found that individuals with obesity frequently provide “blame-absolving narratives to mitigate the negative stereotypes associated with their ‘spoiled' body image” [44, 45]. Health practitioners may benefit from emphasizing the broad reasons for obesity with patients who may feel pressure to explain or justify their struggles with weight. Using the cards may also encourage the inclusion of key family members in helpful discussions. A review of studies that assessed behaviour change in couples concluded that involving a patient's partner in diet treatment programs provided better outcomes, especially in the long term, provided that the partner took on a defined active role [46]. This was also true for physical activity related behaviours. Using the cards in clinical settings may be a good start for soliciting improved conversations and support in the context of home and daily life.

Participants who had been engaged in successful behaviour change for some time reported that they would enjoy using the cards as a monitoring device to remind them of how much progress they had made. This unexpected finding suggests that the process of selecting cards may be used to monitor success with behaviour change over time. At present, changes in weight or BMI are the most common outcome measures used to gauge success in obesity treatment [47]. These measures are more likely to miss progress with obtaining healthy habits that do not directly translate into weight loss. For example, increased physical activity lowers cardiometabolic risk even without any associated weight loss [47, 48]. Experiencing success with behaviour change and receiving education about the benefits of lowering key metabolic risk factors with physical activity may help patients to understand that not all individuals will experience the same rate of weight loss even when taking the same actions. Variations in body shape and size are expected, but sometimes difficult to accept for individuals setting size or weight oriented goals. Using the cards to help track behavioural changes may help patients to shift their health goals and build self-efficacy.

Overall, participants found the cards helpful and expressed the desire to use them in a clinical setting, but they doubted physicians would take the time to talk with them long enough to address their concerns. There is evidence that when family doctors do take the time to engage in behavioural counselling around obesity there are positive outcomes, including weight loss [8, 49] and prevention of weight gain [50]. Use of the cards may help patients to connect better with their physicians, while helping physicians feel there are relevant, tangible goals to work toward collectively.

## Figures and Tables

**Table 1:** Participant characteristics.

Variable	Sample 1 [*n* = 10]	Sample 2 [*n* = 8]
Sex [m/f]	5/5	4/4
Age	51.0 ± 17.6	50.9 ± 13.6
BMI [kg/m^2^]	40.8 ± 8.5	33.7 ± 9.7
Marital status		
Married	3	3
Common law	2	3
Widowed	0	0
Separated	1	1
Divorced	0	1
Single	4	0
Education		
Less than HS	0	0
HS or trade school	4	2
University	3	6
Advanced degree	3	0
Living with others [*y*/*n*]	8/2	6/2
Income		
$0–$25,000	2	2
$26–$50,000	2	0
$51–$60,000	2	1
$61–$80,000	1	1
$80,000+	3	4
Weight loss or diet [*y*/*n*]	8/2	8/0
Medication [*y*/*n*]	9/0	4/4
Chronic disease [*y*/*n*]	9/1	3/5

**Table 2:** Correlation matrix.

		Age	BMI
Number of cards selected as “cards that describe me”	*r*	−0.58*	0.34
	*P* value	0.01	0.19
	*n*	17	17

*Significance at *P* < 0.05.

**Table 3:** Most frequently selected cards.

“Cards that describe me” [*n* = 18]	“Most important cards” [*n* = 18]	Statement
16	1	I have tried dieting and/or weight loss medication.
15	1	I am a very social person.
14	4	**My body size and shape influence how I value myself.**
12	6	**I eat way too quickly.**
10	5	**Sometimes, even when on a diet or not hungry, I eat sensibly in front of others but splurge while I am alone.**
9	5	I never seem to have free time.
9	4	**I often feel helpless and incapable of controlling my urges to overeat and feel desperate to regain control.**

Note: Bolded statements relate to binge eating disorder behaviours.
